# Prediction of the Reference Evapotranspiration Using a Chaotic Approach

**DOI:** 10.1155/2014/347625

**Published:** 2014-07-16

**Authors:** Wei-guang Wang, Shan Zou, Zhao-hui Luo, Wei Zhang, Dan Chen, Jun Kong

**Affiliations:** ^1^State Key Laboratory of Hydrology-Water Resources and Hydraulic Engineering, Hohai University, Nanjing 210098, China; ^2^College of Water Resources and Hydrology, Hohai University, Nanjing 210098, China; ^3^College of Resources & Environmental Sciences, Nanjing Agricultural University, Nanjing 210095, China; ^4^Key Laboratory of Efficient Irrigation-Drainage and Agricultural Soil-Water Environment in Southern China of Ministry of Education, College of Water Conservancy and Hydropower Engineering, Hohai University, Nanjing 210098, China; ^5^College of Harbor, Coastal and Offshore Engineering, Hohai University, Nanjing 210098, China

## Abstract

Evapotranspiration is one of the most important hydrological variables in the context of water resources management. An attempt was made to understand and predict the dynamics of reference evapotranspiration from a nonlinear dynamical perspective in this study. The reference evapotranspiration data was calculated using the FAO Penman-Monteith equation with the observed daily meteorological data for the period 1966–2005 at four meteorological stations (i.e., Baotou, Zhangbei, Kaifeng, and Shaoguan) representing a wide range of climatic conditions of China. The correlation dimension method was employed to investigate the chaotic behavior of the reference evapotranspiration series. The existence of chaos in the reference evapotranspiration series at the four different locations was proved by the finite and low correlation dimension. A local approximation approach was employed to forecast the daily reference evapotranspiration series. Low root mean square error (RSME) and mean absolute error (MAE) (for all locations lower than 0.31 and 0.24, resp.), high correlation coefficient (CC), and modified coefficient of efficiency (for all locations larger than 0.97 and 0.8, resp.) indicate that the predicted reference evapotranspiration agrees well with the observed one. The encouraging results indicate the suitableness of chaotic approach for understanding and predicting the dynamics of the reference evapotranspiration.

## 1. Introduction

Evapotranspiration is one of the most important hydrological variables to consider when estimating hydrologic water balance, performing appropriate water resources allocation, establishing efficient irrigation scheduling and assessing hydrological impact of changing climate conditions [1–4]. Evapotranspiration is affected by some factors including weather parameters, crop characteristics, management, and environmental aspects [5]. Specific devices and accurate measurements of various physical parameters or the soil water balance in lysimeters are required to determine evapotranspiration, and thus direct measurement of evapotranspiration is very difficult for long time and large areas because it is time-consuming and expensive [6]. Reference evapotranspiration (*ET*_0_) represents the effect of the climate on the evapotranspiration process. *ET*_0_ provides an upper limit for actual evapotranspiration and indicates the total available energy in humid and arid climate [7]. Some indirect methods (e.g., [5, 8]) have been proposed for estimating evapotranspiration, and they generally have been developed by multiplying reference evapotranspiration by some correction parameters, such as crop coefficient or soil coefficient [9]. Actual evapotranspiration is a key factor of hydrological cycle and agricultural water management. Therefore, in a sense, the accurate forecast for reference evapotranspiration plays an important role in understanding the hydrological cycle, especially in planning and managing irrigation practices.

Reference evapotranspiration is defined as “the rate of evapotranspiration from a hypothetical reference crop with an assumed crop height of 0.12 m, a fixed crop surface resistance of 70 s m^−1^, and an albedo of 0.23, closely resembling the evapotranspiration from an extensive surface of green grass cover of uniform height, actively growing, and completely shading the ground with adequate water.” Among several existing *ET*_0_ methods, the FAO-56 Penman-Monteith (P-M) method [5] is currently widely used and can be considered as a sort of standard [10]. The P-M method overcomes shortcomings of the previous Penman method. The P-M method provides reference values of potential evapotranspiration for a uniform grass reference surface worldwide and there is no need for local calibration [5]. However, a major drawback to apply the P-M method is its relatively high demand for data. Apart from site location, the method requires air temperature, wind speed, relative humidity, and sunshine duration (or shortwave radiation data). Obtaining large meteorological data sets is difficult, especially in developing countries where the number of the meteorological stations is small and reliable data is scarce [11]. This would lead to the discontinuity for reference evapotranspiration series.

Some new methods for predicting and modeling evapotranspiration are developed or researched considering the limitations and disadvantages of the conventionally applied modeling techniques, such as, simple yearly differencing (YD) model and monthly average (MAV) model [12], seasonal autoregressive integrate moving average (SARIMA) model [13], and transfer function noise (TEN) model [14]. Moreover, in recent years, a large number of researches have been devoted to the application of artificial neural network in the area of modeling and predicting evapotranspiration and reference evapotranspiration (e.g., [15–23]). Most of the methods mentioned above, in fact, still need some basic meteorological values measured in the past as input for the forecast. Few studies have been carried out to forecast reference evapotranspiration using the reference evapotranspiration series alone, in large measure because that reference evapotranspiration can be seen as a complex system impacted by numerous climate factors. The full nonlinear dynamic information for these factors is not easily gained from the reference evapotranspiration series, which impacts on the effects of prediction for reference evapotranspiration.

In recent years, the advent and development of chaos theory provide a new idea for complex systems time series forecast. The study of chaos theory considers that, for a complex system with the chaotic features, a long-term time evolution of any single variable time series from the system contains the evolution information of all the variables in the system. Meanwhile, the self-similarity of reference evapotranspiration has been revealed by fractal analysis by Liu et al. [24] and Xie et al. [25]. Therefore, it is now believed that the dynamic evolution of the seemingly complex hydrological processes such as reference evapotranspiration can be best understood and forecasted using nonlinear deterministic chaotic models; that is, the chaotic method is capable of describing the dynamic changes occurring in the underlying evapotranspiration system using only a single variable time series.

In the past decade, chaos theory was applied in a variety of hydrological processes, such as rainfall (e.g., [26–32]) runoff and discharge (e.g., [28, 33–40]), sediment transport [23, 41], and lake volume [42, 43]. Outcomes of these studies are encouraging, as the chaos method has been found to be very useful in providing important information regarding dynamical characteristics of the various hydrological phenomena and their predictabilities.

It is surprising to note that though chaos theory has widespread applications in hydrology, to the knowledge of the author, no study has been attempted to applying the chaos analysis method to reference evapotranspiration thus far. Recognizing the above concerns, an attempt is made in the present study to understand and forecast the dynamics of daily reference evapotranspiration with chaotic theory from several locations in China.

This paper is organized as follows: the next section of this paper provides a brief description about how to reconstruct a multidimensional phase-space with a scalar time series, followed by a description of the local approximation forecasting approach.  Section 2.3 gives details of the weather stations and data. The results obtained are analyzed and the performance of the chaotic model in forecasting reference evapotranspiration is investigated and discussed in Sections 3 and 4. The summary and concluding remarks are presented in the final section.

## 2. Methods and Materials

### 2.1. Phase-Space Reconstruction Forecasting Approach

Due to joint influence of many factors, a nonlinear system always exhibits intensive complexity. The nonlinear system is so complex that the systematic information from a series may be lost or cannot effectively be extracted, which is usually very useful for systematic evolution. In fact, it is possible to grasp the information about the dynamics of the entire multivariable system by using rational method [41]. The theory describing the evapotranspiration evolution is unknown and hence the dynamics of evapotranspiration cannot be directly determined. A multidimensional phase space from the one-dimensional time series representing the dynamics of the evapotranspiration is reconstructed to map the series to a phase-space trajectory. The basic principles of phase-space reconstruction process are described below. Among a variety of methods available for phase-space reconstruction, the method of delays (e.g., [44]) is the most widely used one.

For a scalar time series *X*_*i*_, where  *i* = 1,2,…, *N*, the phase space can be reconstructed using the method of delays. The basic idea in this method is that the evolution of any single variable of a system is determined by other variables with which it interacts. Information about the relevant variables is thus implicitly contained in the history of any single variable. On the basis of this an “equivalent” phase space can be reconstructed by assigning an element of the time series *X*_*i*_ and its successive delays as coordinates of a new vector time series

(1) $$\begin{array}{c} Y_{j}=\left(X_{j},X_{j+\tau },X_{j+2\tau },\ldots ,X_{j+(m-1)\tau }\right), \end{array}$$

where *j* = 1,2,…, *N* − (*m* − 1)*τ*, *τ* is referred to as the delay time and for a digitized time series is a multiple of the sampling interval used and *m* is the dimension of the vector *Y*_*j*_, called embedding dimension. If the embedding phase-space dimension *m* is large enough, the chaotic attractor, a set of physical properties toward which a system tends to evolve, regardless of the starting conditions of the system, can be depicted.

An appropriate delay time, *τ*, is necessary for the phase-space reconstruction because an optimum selection of *τ* gives best separation of neighboring trajectories within the minimum embedding phase space (e.g., [45, 46]). A too large or too small *τ* would influence the ability of characterizing dynamical system for reconstructed phase space (e.g., [43, 47]).

Many methods have been developed selecting an appropriate delay time. Well known among these are the autocorrelation function method (ACF) (e.g., [48–50]) and the average mutual information method (AMI) (e.g., [51]). The delay time *τ* value is selected as the time when the first zero-crossing of the signal autocorrelation function or *τ* has dropped to 1−1/*e* (*e*, approximately 2.7183 the Euler's constant, which is the base of natural logarithms) of its initial value or where the MI first attains a minimum. No evidence indicates that one of those two methods must be better than the other one [46]; therefore, both of the two methods were adopted in the present study to determinate the delay time of the reference evapotranspiration series.

It is generally believed that there exists a dimension *m* for which the geometric object formed by *Y*_*j*_ is equivalent to the original trajectory *X*_*i*_. A small value of *m* may not adequately reconstruct the original phase space. However, a large value of *m* would reconstruct the original phase but would lead to a high computational cost [52]. Many algorithms have been formulated for the computation of the correlation dimension of a time series, among which Grassberger-Procaccia algorithm [53] is the most popular one and has been successfully applied in a number of studies; therefore, it is employed in the present study too. According to the embedding theorem [44], a (*m* = 2*d* + 1)-dimensional phase space is required to characterize a dynamic system with an attractor dimension *d*. The attractor dimension can be estimated in the following manner. For a *m*-dimensional phase space the correlation function *C*(*r*) is given by

(2) $$\begin{array}{c} C\left(r\right)=\underset{N\to \infty }{\lim}\frac{2}{N\left(N-1\right)}\overset{N}{\underset{i,j=1}{\sum }}H\left(r-\left|Y_{i}-Y_{j}\right|\right), \end{array}$$

where *H* is the Heaviside step function, with *H*(*u*) = 1 for *u* > 0 and *H*(*u*) = 0 for *u* ≤ 0, where *u* = *r* − |*Y*_*i*_ − *Y*_*j*_|, *r* is the radius of sphere centered on *Y*_*i*_ or *Y*_*j*_, and *N* is the number of data points. If the time series is characterized by an attractor, then for positive values of *r* the correlation function *C*(*r*) is related to the radius *r* by the following relation:

(3) $$\begin{array}{c} C\left(r\right)\propto \alpha r^{D_{2}}, \end{array}$$

where *α* is a constant and *D*_2_ is the slope or the correlation exponent of ln⁡*C*(*r*) versus ln⁡*r* plot given by:

(4) $$\begin{array}{c} D_{2}=\underset{r\to 0}{\lim}\frac{\ln C\left(r\right)}{\ln r} \end{array}$$

The slope is generally estimated by a least squares fit of a straight line over a certain range of *r*, called the scaling region. For a finite data set, such as the reference evapotranspiration data series, *r* cannot get too close to zero. To handle this situation, a better way of identifying the scaling region is to plot ln⁡*C*(*r*) versus ln⁡*r* and select the apparently linear portion of the graph. The slope of this portion will approximate *D*_2_, to observe whether chaos exists, and the correlation exponent values are plotted against the corresponding embedding dimension values. If the value of the correlation exponent *D*_2_ is finite, low and noninteger, then the system is considered to exhibit low-dimensional chaos, and the saturation value of the correlation exponent is defined as the correlation dimension of the attractor. On the contrary, if the correlation exponent *D*_2_ increases without bond with increase in the embedding dimension *m*, then the system under investigation is considered as stochastic.

Many prediction methods based on phase space reconstruction have been developed, and the local approximation method successfully applied to hydrological time series forecast [23, 41, 54] is used here and is explained as follows.

The phase space of the time series *X*_*i*_ can be reconstructed to vector *Y*_*j*_ (described in this section), and a functional relationship between the vectors *Y*_*j*_ can be assumed, such as

(5) $$\begin{array}{c} Y_{j+T}=f_{T}\left(Y_{j}\right), \end{array}$$

where *Y*_*j*_ and *Y*_*j*+*T*_ are vectors of dimension *m*, describing the state of the system at times *j* (e.g., current state) and *j* + *T* (e.g., future state), respectively. The problem then is to find an appropriate expression for *f*_*T*_ (e.g., the predictor *F*_*T*_).

There are many approaches to obtain *F*_*T*_, according to the way for approximation, which can be broadly divided into global approximation approach and local approximation approach. In global approximation a function *F*_*T*_ valid over the entire state space is considered. On the other hand, the local approximation approach subdivides the domain of the attractor into many subsets, each of which is represented by an approximation *F*_*T*_ valid only in that same subset. Since the local approximation approach can lead to a considerable reduction in the complexity of the representation *F*_*T*_ without degrading the quality of the forecast, it has been considered that, for a very short term, the local approximation can generally provide better results than those obtained using the global approximation approach [41]. Therefore, the local approximation approach is employed in this study. Local polynomial technique, widely used local approximator, is described in detail as follows.

The identification of the sets in which to subdivide the domain is done by fixing a metric || || and then, given the starting *Y*_*j*_ from which the forecast is initiated, identifying neighbors *Y*_*j*_^*p*^, which constitute the set corresponding to *Y*_*j*_ is done. Based on this, the local functions can be constituted. The next neighborhood is *Y*_*j*+1_^*p*^. A local polynomial model of this evolution can be expressed by [41]

(6) $$\begin{array}{c} Y_{j+1}^{p}=A+BY_{j}^{p}+C{\left(Y_{j}^{p}\right)}^{2}, \end{array}$$

where *A*, *B*, and *C* are constants, which are learned from the training sets. After the nearest neighbors are found, each of these states can be projected to their respective future states and a local predictor can be constructed by a least squares fit minimizing

(7) $$\begin{array}{c} \overset{\text{NB}}{\underset{p=1}{\sum }}{||Y_{j+1}^{p}-F_{j}\left(Y_{j}^{p}\right)||}^{2}, \end{array}$$

where *Y*_*j*_^*p*^, *p* = 1,2,…, NB, are the nearest neighbors of *Y*_*j*_ and *Y*_*j*+1_^*p*^ are their corresponding one-step ahead states. In the present study, the local maps are learned using local polynomials (e.g., [42]). Based on those local maps, the forecasts are made from a new point *Z*_0_ and the nearest neighbor in training set *Y*_*q*_ is found. Consequently, the one-step evolution of *Z*_0_ is found that is denoted as *Z*_1_ and given by

(8) $$\begin{array}{c} Z_{1}=F_{q}\left(Z_{0}\right). \end{array}$$

And then the nearest neighbor to *Z*_1_ is found and the procedure is repeated until the desired prediction horizon is reached.

### 2.2. The FAO Penman-Monteith Method

The P-M method for calculating daily reference evapotranspiration can be summarized as follows [5]

(9) $$\begin{array}{c} {ET}_{0}=\frac{0.408\Delta \left(R_{n}-G\right)+\gamma \left(900/\left(T+273\right)\right)\mu_{2}\left(e_{s}-e_{a}\right)}{\Delta +\gamma \left(1+0.34\mu_{2}\right)}, \end{array}$$

where *R*_*n*_ is the net radiation at the crop surface (MJ m^−2^ day^−1^), *G* is the soil heat flux density (MJ m^−2^ day^−1^), *T* is the mean daily air temperature at 2 m height (°C), *u*_2_ is the wind speed at 2 m height (m s^−1^), *e*_*s*_ is the saturation vapor pressure (kPa), *e*_*a*_ is the actual vapor pressure (kPa), *e*_*s*_ − *e*_*a*_ is the saturation vapor pressure deficit (kPa), Δ is the slope of the vapor pressure (kPa°C^−1^), and *γ* is the psychrometric constant (kPa°C^−1^).

A complete set of equations to compute the parameters of the above equation and all the data required for the calculation of the reference evapotranspiration are given in [5, chapter 3 in the FAO paper 56].

### 2.3. Study Area and Data

**Figure 1:**
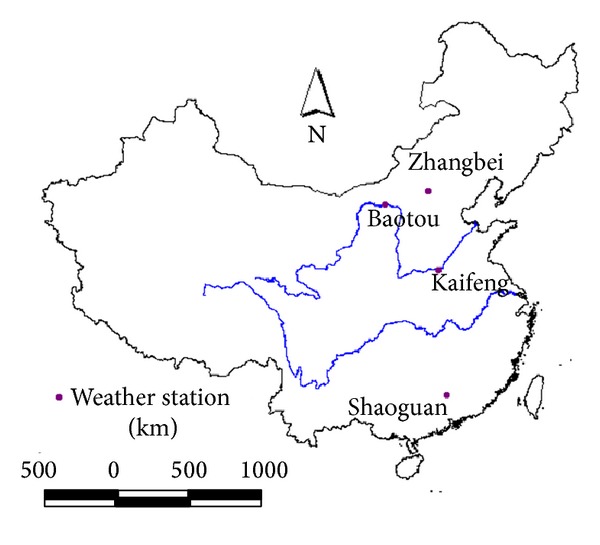
Locations of the weather stations used in this study.

Data used in this study from four National Meteorological observatory (NMO) stations (i.e., Baotou, Zhangbei, Kaifeng, and Shaoguan) is provided by the National climatic Centre (NCC) of CMA (the China Meteorological Administration), including daily observations of maximum, minimum and mean air temperature, wind speed, relative humidity, and sunshine duration for the period of 40 years (from 1966 to 2005). In terms of different latitudes and longitudes, the spatial distribution of stations selected covers a wide range of climatic region in China. The respective locations are shown in Figure 1 and described in Table 1. The FAO56 P-M method was the sole method recommended by FAO to calculate reference evapotranspiration wherever the required input data are available. In the present study, therefore, *ET*_0_ computed using the FAO56 P-M method from observed weather variables is taken as the standard.

**Figure 2:**
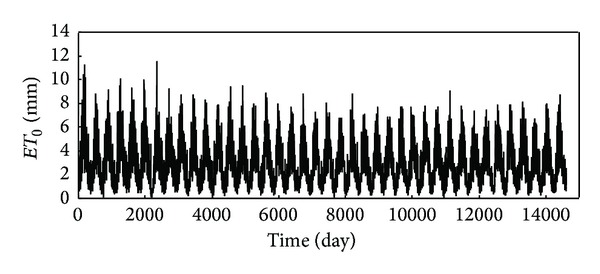
Variation of daily reference evapotranspiration (January 1966–December 2005) from Kaifeng station.

For each of the four locations, the reference evapotranspiration data computed by FAO56 P-M method consisted of 40 years' (1966–2005) daily data records.  Table 2 gives some of the important statistics of the daily reference evapotranspiration series computed by FAO56 P-M method from the observed weather variables between 1 January, 1966, and 31 December, 2005. As an example, the variation of reference evapotranspiration series from Kaifeng station is shown in Figure 2. With presenting an evident annual and/or seasonal cycle, Figure 2 clearly indicates that the reference evapotranspiration series obviously present significant variation at the same time. Whether the stochastic or chaotic behaviour cannot be directly identified by the seemingly irregular behavior of the reference evapotranspiration series. Therefore, other methods are required to identify whether or not chaos exists. Similar results are also made for the reference evapotranspiration series of Baotou station, Shaoguan station, and Zhangbei station. The data samples consist of 40 years (1966–2005) of daily reference evapotranspiration data computed by FAO56 P-M method. Each data set is divided into two parts: the first part of the series is used to investigate the existence of chaos, to find the appropriate embedding parameters in the phase space and to reconstruct the phase space, where the delay time and the correlation dimension are calculated; the forecast is performed on the other part. Therefore, in the present study, the entire data set of 40 years (January 1966–December 2005) is split into two parts: the first 39 years (January 1966–December 2004) of data are used in training and learning (i.e., the phase-space reconstruction) and predictions are made for the subsequent 1 year (January 2005–December 2005) of data.

## 3. Results

### 3.1. Estimation of the Reconstruction Parameters

**Figure 3:**
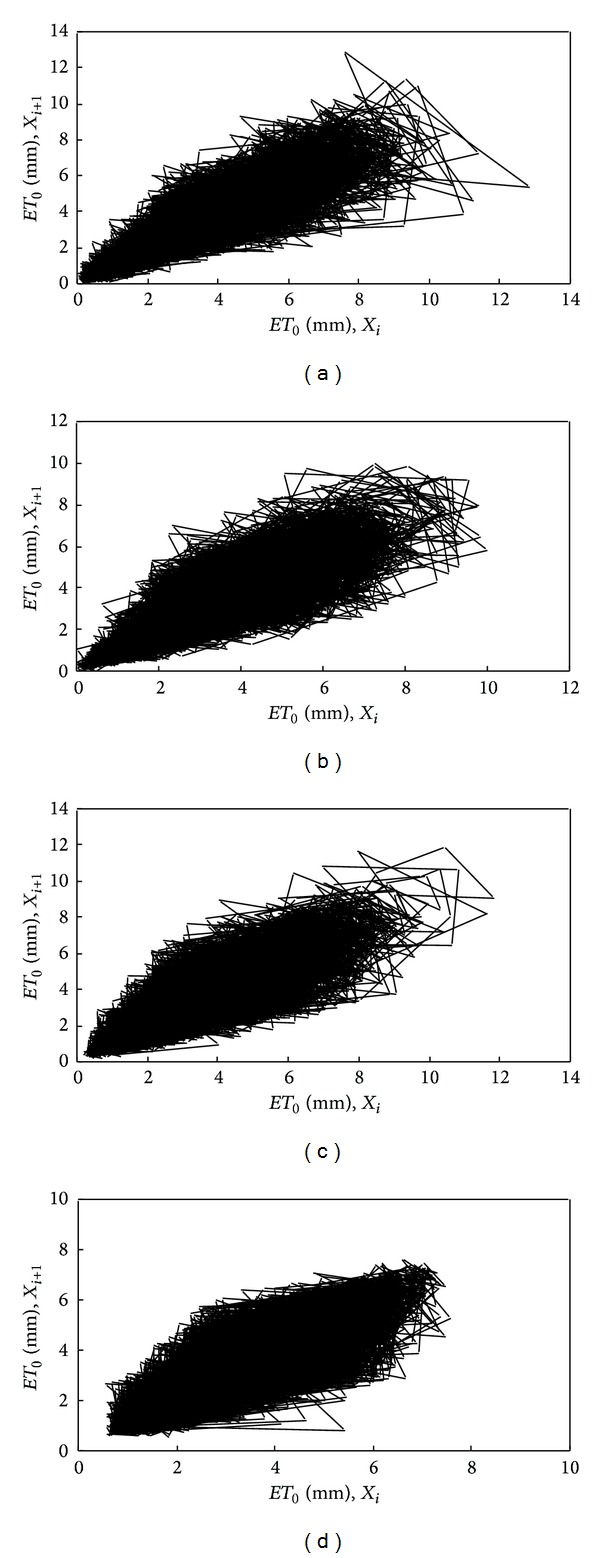
Two-dimensional phase portrait of the daily reference evapotranspiration time series reconstructed for *τ* = 1: (a) Baotou station; (b) Zhangbei station; (c) Kaifeng station; (d) Shaoguan station.

Figure 3 shows the feature of the two-dimensional phase of the reconstructed attractor for the reference evapotranspiration series from Baotou, Shaoguan, Kaifeng, and Zhangbei, respectively, with delay time *τ* = 90, that is, the projection of the attractor on the plane {*X*_*i*_, *X*_*i*+1_}.

How the series of the scalar reference evapotranspiration is reconstructed in a higher dimensional phase space was illustrated in Figure 3. It can be clearly seen from Figure 3 that a well-defined attractor can be reconstructed for all the stations, except a very few outliers corresponding to some very high values of the reference evapotranspiration series. This implied that the feature of the dynamic changes of the seemingly highly irregular reference evapotranspiration can be seen as a relatively simple evolution using the trajectories of the phase-space diagram.

**Figure 4:**
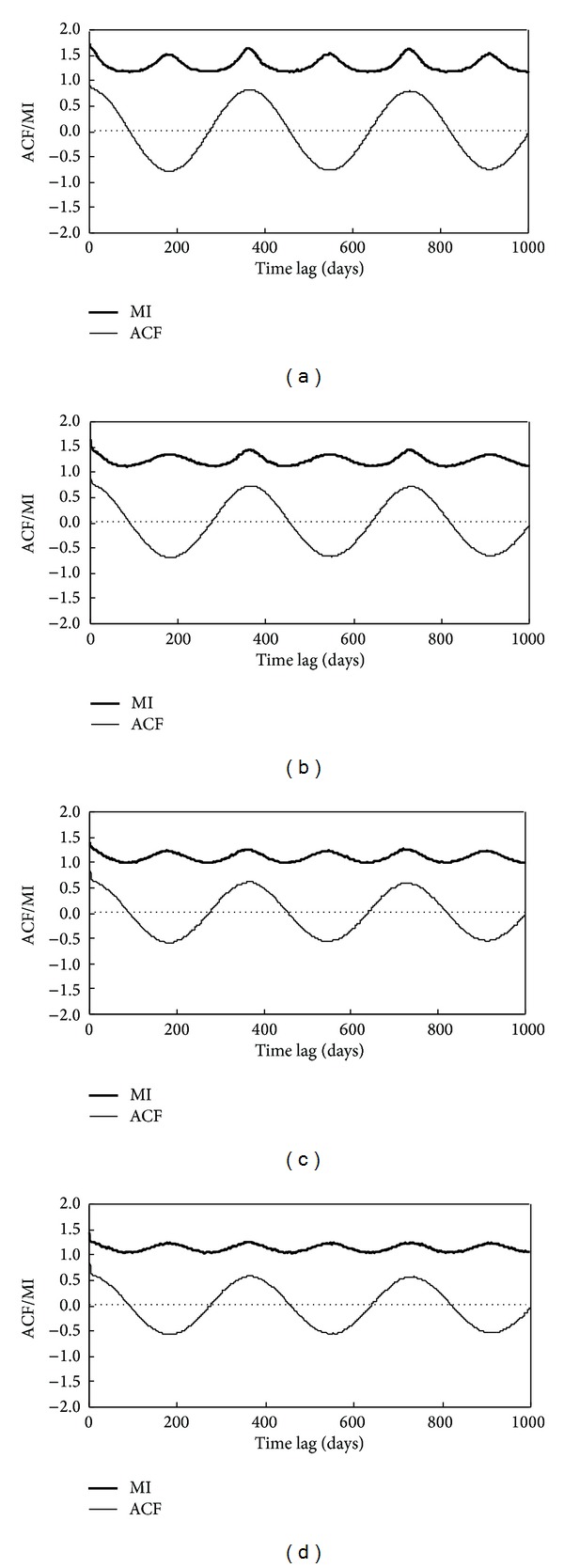
ACF and MI of daily reference evapotranspiration: (a) Baotou station; (b) Zhangbei station; (c) Kaifeng station; (d) Shaoguan station.

In order to reconstruct the original phase space, the appropriate delay time *τ* and embedding dimension *m* should be estimated firstly. ACF and MI for the reference evapotranspiration were calculated, which were shown in Figure 4. Because of strong seasonality, ACF first attains zeros at the lag time of about 1/4 period, namely, 91, 89, 91, and 91 days for Baotou, Zhangbei, Kaifeng, and Shaoguan, respectively. The MI method gives similar estimation for delay time  *τ*  to the ACF method, about approximately 1/4 annual period. The detailed delay time *τ* values are 91, 89, 90, and 90 days for Baotou, Zhangbei, Kaifeng, and Shaoguan, respectively. From the delay time results calculated by ACF method and MI method, we can conclude that for the reference evapotranspiration series it really does not matter whether the autocorrelation function or the mutual information is used.

**Figure 5:**
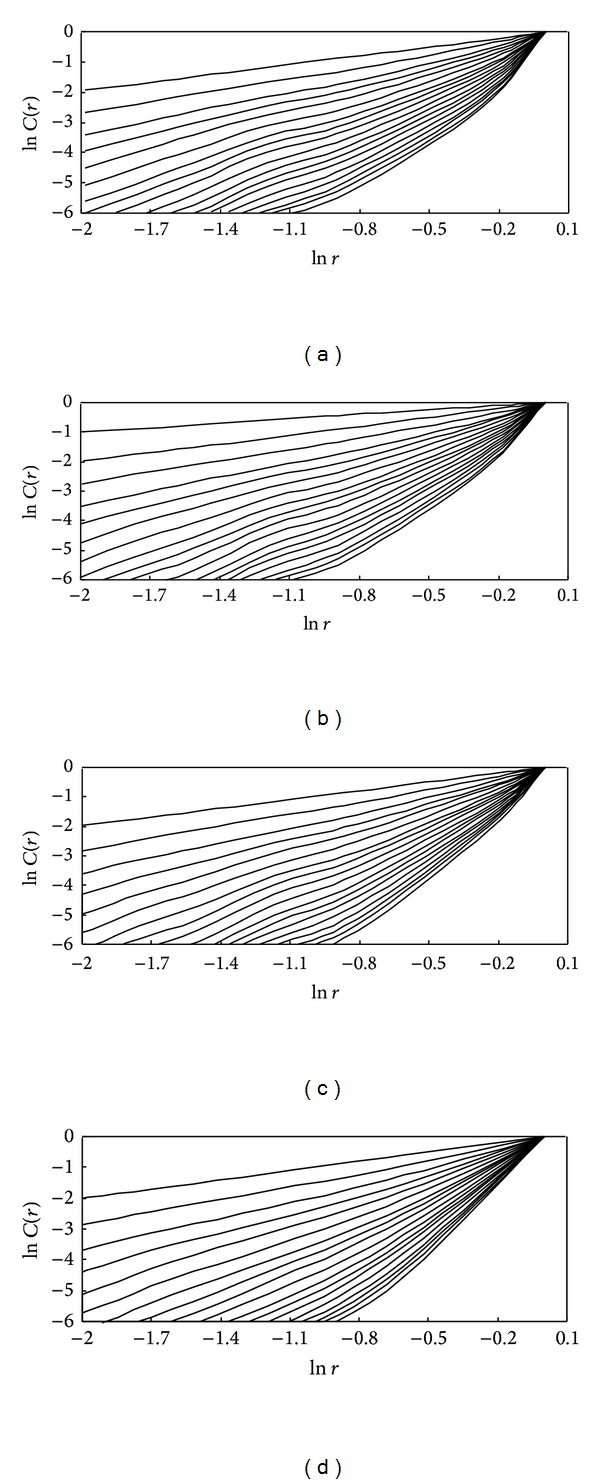
ln⁡*C*(*r*) versus ln⁡*r* plots for daily reference evapotranspiration series: (a) Baotou station; (b) Zhangbei station; (c) Kaifeng station; (d) Shaoguan station.

**Figure 6:**
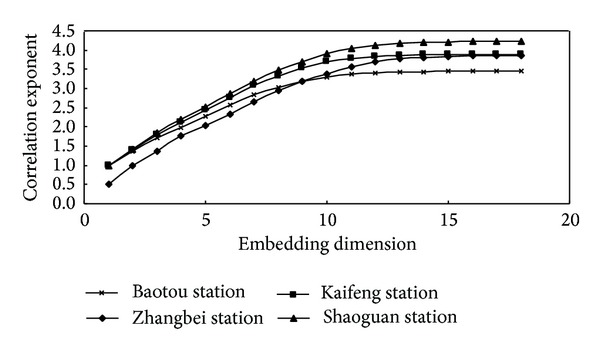
Relationship between correlation exponent and embedding dimension for daily reference evapotranspiration series.

In the present study, the correlation function and hence the exponents are computed using the delay times determined by the MI method in the previous section, as explained in Section 2.1, for the reference evapotranspiration series. The delay time values of 91, 90, 91, and 89 are obtained for Baotou, Zhangbei, Kaifeng, and Shaoguan, respectively. Correlation dimension method is a common algorithm to determine the optimum embedding dimension and the correlation dimension, meanwhile, correlation dimension method is a method to investigate the chaotic behavior of the time series by identifying whether the correlation exponent value can “become saturation” with the increased embedding dimension. For the reference evapotranspiration time series from the four stations studied, the relationship between the correlation integral, *C*(*r*), and the radius, *r* (i.e., ln⁡*C*(*r*) versus ln⁡*r*), for embedding dimensions, *m*, from 1 to 18, was shown in Figure 5. Figure 5 indicates explicit scaling regions that allow fairly accurate estimates of the correlation exponents.  Figure 6 shows the relationship between the correlation exponent values and the embedding dimension values for the four stations. It can be seen that, for the reference evapotranspiration time series from Baotou, Zhangbei, Kaifeng, and Shaoguan, the correlation exponent value increases with the embedding dimension up to 12, 12, 13, and 12, respectively, and then becomes saturation beyond these values. The saturation of the correlation exponent beyond a certain embedding dimension value is an indication of the existence of deterministic dynamics [32, 52]. The finite and low correlation dimensions are observed in all the cases, indicating that the reference evapotranspiration time series from the four stations exhibit low-dimensional chaotic behaviors. The correlation dimension results achieved for the reference evapotranspiration time series from different stations are listed in Table 3.

A data set with high variability in the values provides a high dimension and indicates the higher complexity of the dynamics of the phenomenon. The dimension results obtained in the present study indicate that the reference evapotranspiration from Shaoguan station has higher variability and irregularity than those from other stations. The nearest integer above the saturation value provides the minimum number of phase spaces or variables necessary to model the dynamics of the attractor. Taking the reference evapotranspiration time series from Shaoguan station as an example, the correlation dimension value about 4.13 indicates that the minimum number of variables necessary to characterize the dynamic feature of the reference evapotranspiration is 5. For other stations in the present study, the minimum number of variables necessary to characterize the dynamic feature of reference evapotranspiration shown in Table 3 all is 4.

### 3.2. Prediction Results with Chaotic Method

In view of the above analysis, it should be clear that the dynamics of reference evapotranspiration from all the stations under different climates in China can be well described by a chaotic approach. Namely, there are the possibilities of achieving good forecasts for the reference evapotranspiration series, especially with the use of local approximation methods.

The local approximation method explained in Section 2.1 is now employed to forecast the reference evapotranspiration series from the four selected stations in the present study. The delay time and optimum embedding dimensions calculated in the previous section for the reference evapotranspiration series from each station are used for reconstructing the phase space, and predictions are made for one time step ahead (i.e., lead time = 1). The performances of the local approximation prediction method are evaluated using some statistical indices including mean absolute error (MAE), root mean square error (RMSE), correlation coefficient (CC), and modified coefficient of efficiency. The detailed definitions of these error indicators (absolute and relative) are described as follows:

(a) Mean absolute error (MAE) is expressed by

(10) $$\begin{array}{c} \text{MAE}=\frac{\sum_{i=1}^{N}\left|O_{i}-P_{i}\right|}{N}. \end{array}$$

(b) The root mean square error (RMSE) is expressed by

(11) $$\begin{array}{c} \text{RMSE}=\sqrt{\frac{\sum_{i=1}^{N}{\left(P_{i}-O_{i}\right)}^{2}}{N}}, \end{array}$$

where *N* is the number of observations and *O*_*i*_ and *P*_*i*_ are, the *i*th observed (calculated with the FAO56 P-M method) and predicted data (using the local approximation prediction method).

(c) Correlation coefficient (CC) is expressed by

(12) $$\begin{array}{c} r=\frac{\sum_{i=1}^{N}\left(O_{i}-\bar{O}\right)\left(P_{i}-\bar{P}\right)}{{\left[\sum_{i=1}^{N}{\left(O_{i}-\bar{O}\right)}^{2}\right]}^{1/2}{\left[\sum {\left(P_{i}-\bar{P}\right)}^{2}\right]}^{1/2}}, \end{array}$$

where $\bar{O}$ and $\bar{P}$ are the mean of the observed and predicted data in the evaluation set, respectively.

(d) Modified coefficient of efficiency is

(13) $$\begin{array}{c} E1=1-\frac{\sum_{i=1}^{N}\left|O_{i}-P_{i}\right|}{\sum_{i=1}^{N}\left|O_{i}-\bar{O}\right|}. \end{array}$$

The correlation coefficient ranges from 0 to 1, with higher values indicating better agreement between the observed and the predicted data, whereas the modified coefficient of efficiency ranges from −*∞* to 1, with higher positive values indicating better agreement.

**Figure 7:**
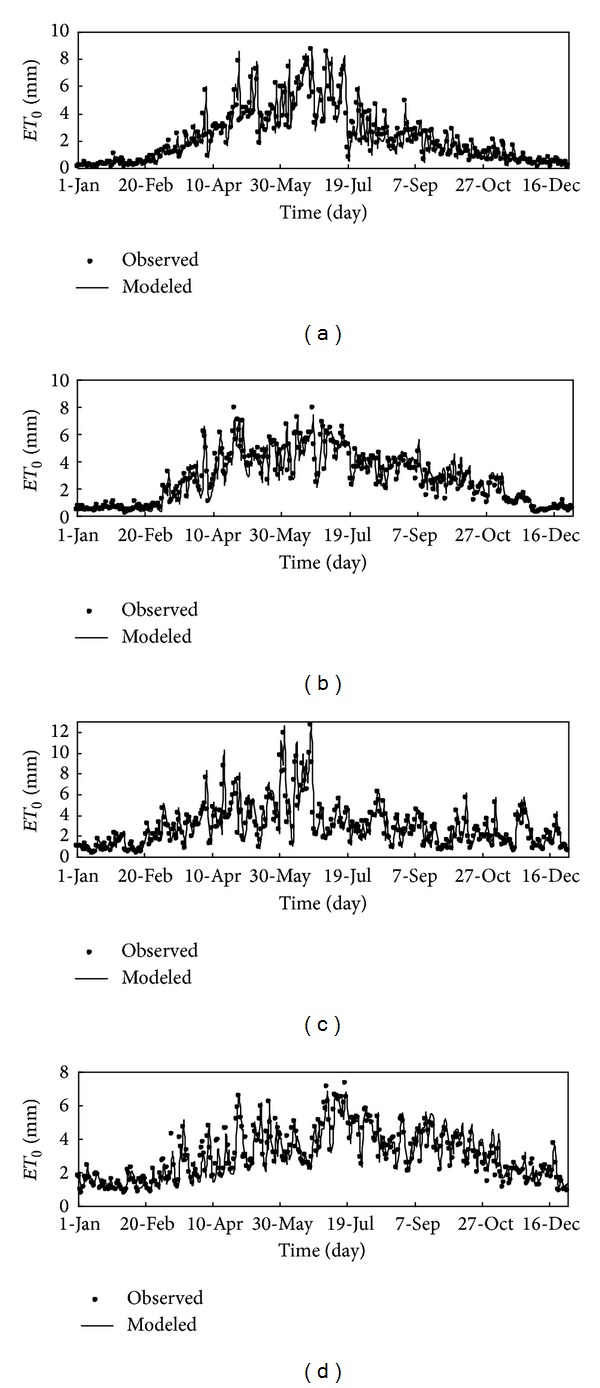
Comparison of observed and predicted daily reference evapotranspiration: (a) Baotou station; (b) Zhangbei station; (c) Kaifeng station; (d) Shaoguan station.

**Figure 8:**
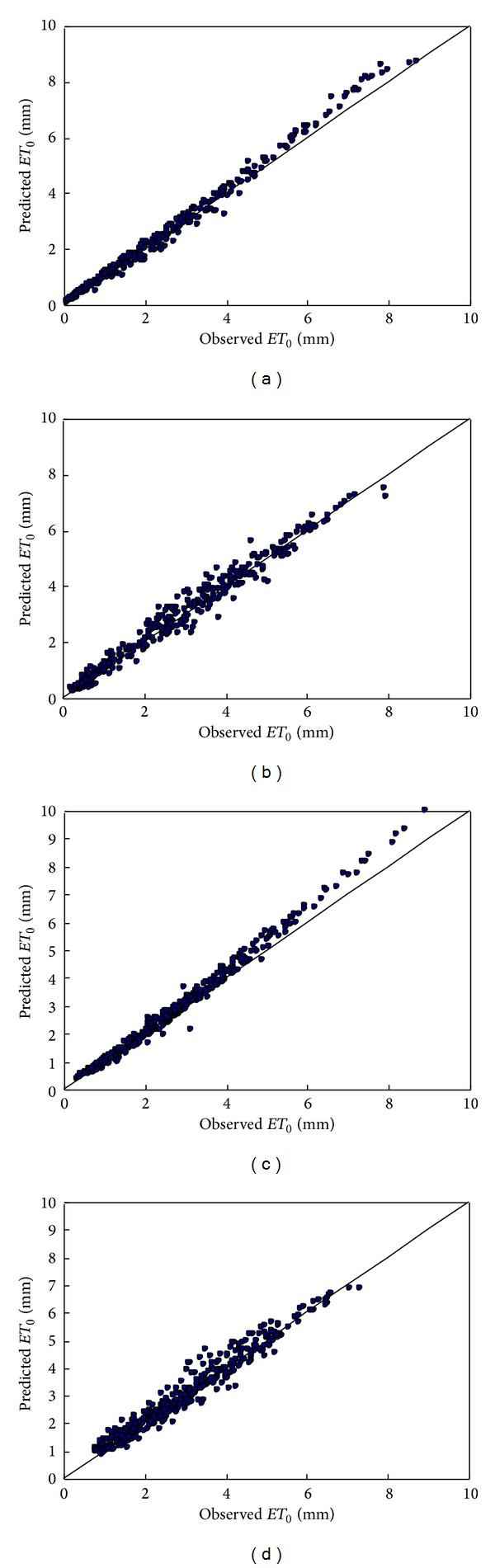
Scatter plot of daily *ET*_0_ series forecasted by chaotic model and that computed with observed weather variables: (a) Baotou station; (b) Zhangbei station; (c) Kaifeng station; (d) Shaoguan station.

Figure 7 presents a comparison of the reference evapotranspiration time series for all the stations between the modeled values and the observed values. The scatter plot of the modeled and the observed values is presented in Figure 8, with the solid 1 : 1 (diagonal) line for reference.

Table 4 presents a summary of forecasted results, in terms of MAE, RMSE, CC, and modified coefficient of efficiency (*E*1), for each of the four stations in this study using the chaotic model.

**Figure 9:**
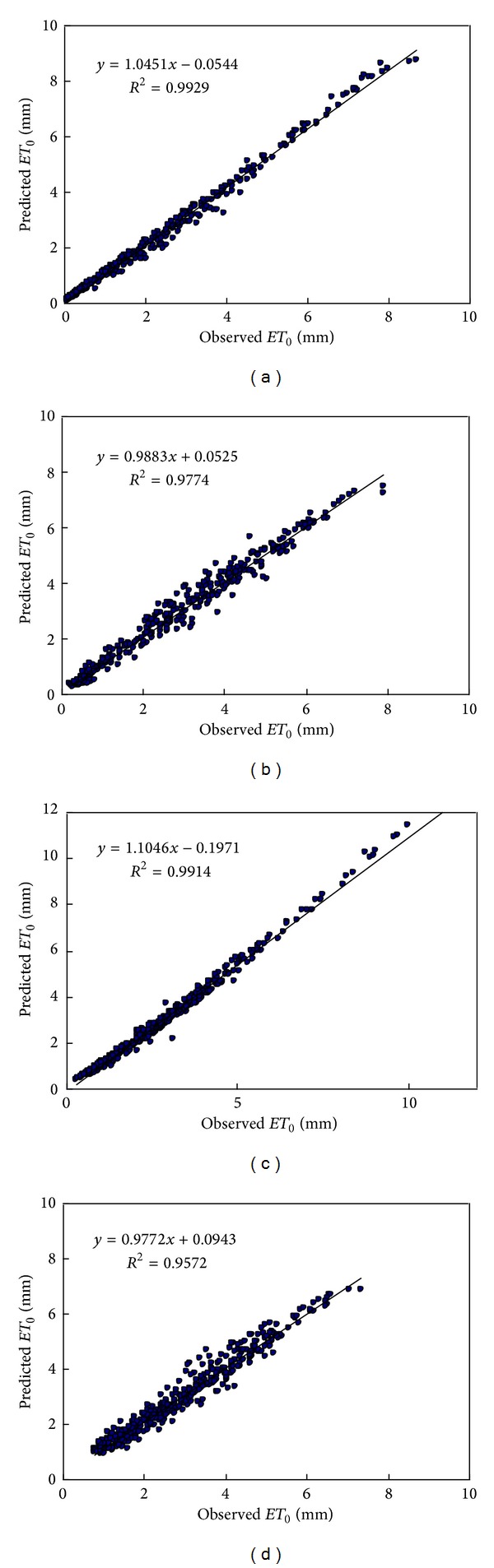
The regression relationship between daily *ET*_0_ forecasted by chaotic model and that computed with observed weather variables: (a) Baotou station; (b) Zhangbei station; (c) Kaifeng station; (d) Shaoguan station.

In respect to each station, these results are the best results among the ones achieved with different number of neighbors for the optimal embedding dimension. For each station, the number of neighbors that yields the best results, which can be also called the optimum number of neighbors, is also presented in Table 4. The statistics in Table 4 indicate that reasonably good forecasts are achieved for all the weather stations selected in the present study. Results for CC and *E*1 values are larger than 0.97 and 0.8 for all the stations, respectively. All MAE and RMSE values are smaller than 0.35 mm day^−1^ and 0.25 mm day^−1^. A closer look at the statistics shows the best and the relative bad predictions are achieved (in terms of the four evaluation indices) in Baotou station (with CC = 0.9965, RMSE = 0.1999, MAE = 0.1378, *E*1 = 0.9130) and Shaoguan station (with CC = 0.9784, RMSE = 0.3054, MAE = 0.2358, *E*1 = 0.8078). A comparison of the observed values with the predicted ones in the form of time series plots is presented in Figure 7. As can be seen, the predicted values are, in general, in good agreement with the observed one for all the four stations. A closer look at the fig reveals that the local approximation method can capture very well the major trends and minor fluctuations in the reference evapotranspiration series and even most of extreme values are fairly well predicted except a few very high values (reference evapotranspiration in summer) at Baotou station and Kaifeng station. In addition, the performance mentioned above of the chaotic model can be revealed by the scatter plots of forecasted and observed reference evapotranspiration series shown in Figure 8, where the solid 1 : 1 line is plotted for reference. The regression relationship between the forecasted and observed reference evapotranspiration series at all the stations was shown in Figure 9. Also, the reference evapotranspiration series values forecasted by chaotic model obviously agree well with those computed with full-observed meteorological data sets. The variance of residuals of the regression is about the same for high, middle, and low values for Baotou and Kaifeng stations, but it is smaller for those two stations (i.e., arid and semihumid location) than the other two stations, Shaoguan and Zhangbei stations (i.e., semiarid and humid location). Moreover, for comparison, autoregressive (AR) model and back propagation (BP) neural network model are also employed in this study to forecast the reference evapotranspiration. The three network layers are adopted in BP neural network model. The input variables include daily mean temperature, sunshine duration, relative humidity, and wind speed in 2 m. The output is daily *ET*_0_. The summary of forecasted results by AR model and BP neural network model is also presented in Table 4. It can be apparently seen that the results by chaotic model are similar to that calculated by BP neural network model based on weather data, but it is better than that simulated by AR model. This encouraging result indicates that the chaos theory provides a new method for *ET*_0_ prediction without weather data.

## 4. Discussion

Reference evapotranspiration is a complex variable influenced by a lot of factors, with a significant system dynamic feature, and the suitability of the phase-space reconstruction and local approximation prediction approaches for understanding those dynamics is indicated by the accurate prediction results measured via the statistic indices, the time series plots, the scatter diagrams, and the regression relationship for the forecasted and observed values. The minimum number of variable necessary to describe the dynamics of the reference evapotranspiration can be determined by the correlation dimension values computed by the correlation dimension method. In the present study, the correlation dimension results achieved for the four stations are 3 or 4 (Table 3), which means that the reference evapotranspiration dynamics is dominantly influenced by 4 or 5 variables. The results are similar to the research for other hydrological series with the chaos theory; for example, only three variables dominantly influenced the suspended sediment concentration dynamics, even though a large number of variables may have influence on its dynamics [41]. What are these 4 or 5 variables? The well-known facts are that the several important impact factors of the reference evapotranspiration are temperature, air humidity, wind speed, and sunshine hours (or solar radiation). With regard to the present results, a possible implication is that temperature, air humidity, wind speed, and sunshine hours are exactly the 4 or 5 variables achieved by the correlation dimension approach. Nevertheless, a further confirmation and verification still should be made.

In any case, a multivariable time series phase-space reconstruction for a single variable time series can recover the dynamics of reference evapotranspiration, which have been echoed by the results that reference evapotranspiration series can be well forecasted by chaotic model.

Different from the reported reference evapotranspiration forecast methods, which are established based on the statistics of the time series variation, such as yearly differencing (YD) or monthly average model (MAV) [12], the seasonal autoregressive integrated moving average model (SARIMA) [16], transfer function noise (TFN) [14], and ANN method [16], chaotic forecast model is based on the representation of the reference evapotranspiration dynamics in the reconstructed phase-space step by step in local neighborhoods.

## 5. Summary

The analytical and forecasted method, based on the chaos theory, used to understand and predict the dynamics of reference evapotranspiration series, was tested for four weather stations (namely, Baotou, Zhangbei, Kaifeng, and Shaoguan) located at different latitudes and longitudes with different climates. The studied reference evapotranspiration time series of the four weather stations is over a period of 40 years (January 1966–December 2005), which is calculated by FAO56 Penman-Monteith model with the daily weather data. The delay time of the reference evapotranspiration series was first estimated with both the zero-crossing of the autocorrelation function and the first minimum of the mutual information. For representing the dynamics of the reference evapotranspiration, a multidimensional phase space was reconstructed for the scalar reference evapotranspiration series. The correlation dimension method was used to investigate the presence of chaos in the reference evapotranspiration series at the four locations selected in this study, and then a local approximation method was employed for making predictions of the reference evapotranspiration.

The results have shown that chaotic characteristics obviously exist in the reference evapotranspiration series at the four different locations (i.e., Baotou, Zhangbei, Kaifeng, and Shaoguan), duo to the finite and low correlation dimension (i.e., 3.41, 3.76, 3.83, and 4.13, resp.). The good performance for the chaotic approach to forecast the reference evapotranspiration series was presented by the accurate predictions for the four locations and measured via statistics indices (i.e., low RSME and MAE; high CC and modified coefficient of efficiency values for all the four stations), suggesting the effectiveness of the chaotic approach for reference evapotranspiration forecast. These results indicated that, seemingly, the variation of the reference evapotranspiration is irregular, in fact, which can be seen as the result of a simple deterministic system influenced by several dominant variables, the number of which determined by the correlation dimension value of the series. The correlation dimension value are 4, 4, 4, and 5, respectively, for Baotou, Zhangbei, Kaifeng, and Shaoguan. In regard to this, those dominant variables may be the temperature, relative humidity, wind speed, and solar radiation, which are considered as the important factors based on physical mechanism.

To the knowledge of the author, this study was the first ever study conducted on the analysis of reference evapotranspiration series based on the chaos theory. However, the present investigation is only the first step to lead to more complete understanding of the dynamics of the hydrological processes for the reference evapotranspiration. Our attempt should continue by applying the chaos concept to time series of the physical factors of the reference evapotranspiration, such as temperature, relative humidity, solar radiation, elevation, and wind speed, which may allow us to provide general and strong conclusion regarding the use of such a concept, for example, the physical explanation to the chaos phenomenon. Moreover, comparing the chaotic approach with other existing forecast approaches to determine a better approach for the evapotranspiration system is the future work. In a word, the authors believe that the present study is a beneficial attempt in these directions, and the further studies in this field are under way and will be addressed in forthcoming papers.

## Figures and Tables

**Table 1:** Locations of weather stations under different climates.

Station	Latitude	Longitude	Elevation (m)	Climate
Baotou	40.667°N	109.850°E	1067.2	Arid
Zhangbei	41.150°N	114.700°E	1393.3	Semiarid
Kaifeng	34.783°N	114.300°E	73.7	Semihumid
Shaoguan	24.683°N	113.600°E	61	Humid

**Table 2:** Statistics of daily reference evapotranspiration data from different stations selected in the present study.

Parameter	Baotou	Zhangbei	Kaifeng	Shaoguan
Mean	3.02	2.77	3.01	3.00
Maximum value	12.83	9.99	11.82	7.57
Minimum value	0.02	0.01	0.19	0.54
Skew	0.51	0.56	0.64	0.60
Standard deviation	2.09	1.84	1.84	1.49
Variance	4.38	3.40	3.39	2.22
Data length	14610	14610	14610	14610

**Table 3:** Results of reconstruction parameters of reference evapotranspiration series.

Parameter	Baotou	Zhangbei	Kaifeng	Shaoguan
Delay time	91	91	91	91
Embedding dimension	12	13	12	12
Correlation dimension	3.41	3.76	3.83	4.13
Number of variables	4	4	4	5

**Table 4:** Prediction results for daily reference evapotranspiration of the stations selected in China by chaotic model, AR model, and BP neural network model.

	Location	Mean absolute error (MAE)	Root mean square error (RMSE)	Correlation coefficient (CC)	Modified coefficient of efficiency	Optimal embedding dimension (m)	Optimal number of neighbors
Chaotic Model	Baotou	0.1378	0.1999	0.9965	0.913	4	72
	Zhangbei	0.2023	0.2799	0.9887	0.8709	4	76
	Kaifeng	0.1962	0.3095	0.9957	0.8679	4	80
	Shaoguan	0.2358	0.3054	0.9784	0.8078	4	68

BP Model	Baotou	0.203	0.098	0.992	0.872		
	Zhangbei	0.189	0.089	0.989	0.894		
	Kaifeng	0.198	0.076	0.997	0.871		
	Shaoguan	0.209	0.082	0.993	0.834		

AR Model	Baotou	0.573	0.665	0.876	0.652		
	Zhangbei	0.612	0.672	0.889	0.673		
	Kaifeng	0.553	0.589	0.897	0.709		
	Shaoguan	0.557	0.699	0.903	0.655		
